# Functional Grading of Mycelium Materials with Inorganic Particles: The Effect of Nanoclay on the Biological, Chemical and Mechanical Properties

**DOI:** 10.3390/biomimetics7020057

**Published:** 2022-05-05

**Authors:** Elise Elsacker, Lars De Laet, Eveline Peeters

**Affiliations:** 1 Architectural Engineering Research Group, Department of Architectural Engineering, Vrije Universiteit Brussel, Pleinlaan 2, B-1050 Brussels, Belgium; lars.de.laet@vub.be; 2 Research Group of Microbiology, Department of Bioengineering Sciences, Vrije Universiteit Brussel, Pleinlaan 2, B-1050 Brussels, Belgium

**Keywords:** mycelium-based composites, lignocellulosic fibers, natural fiber reinforcement, mechanical characteristics, manufacturing variables, nanoclay

## Abstract

Biological materials that are created by growing mycelium-forming fungal microorganisms on natural fibers can form a solution to environmental pollution and scarcity of natural resources. Recent studies on the hybridization of mycelium materials with glass improved fire performance; however, the effect of inorganic particles on growth performance and mechanical properties was not previously investigated. Yet, due to the wide variety of reinforcement particles, mycelium nanocomposites can potentially be designed for specific functions and applications, such as fire resistance and mechanical improvement. The objectives of this paper are to first determine whether mycelium materials reinforced with montmorillonite nanoclay can be produced given its inorganic nature, and then to study the influence of these nanoparticles on material properties. Nanoclay–mycelium materials are evaluated in terms of morphological, chemical, and mechanical properties. The first steps are taken in unravelling challenges that exist in combining myco-fabrication with nanomaterials. Results indicate that nanoclay causes a decreased growth rate, although the clay particles are able to penetrate into the fibers’ cell-wall structure. The FTIR study demonstrates that *T. versicolor* has more difficulty accessing and decaying the hemicellulose and lignin when the amount of nanoclay increases. Moreover, the addition of nanoclay results in low mechanical properties. While nanoclay enhances the properties of polymer composites, the hybridization with mycelium composites was not successful.

## 1. Introduction

In recent years, the exciting characteristics of filamentous fungi did not go unnoticed in the context of biodegradable materials, providing a low-cost and environmentally sustainable solution compared to the production and life cycle of petroleum-based materials [1,2,3,4,5,6,7]. These composite materials are realized by growing the fungi into lignocellulosic fibers, thereby valorizing organic waste streams, and generating dense materials with a construction material application [8]. Typically, composites are composed of a matrix and a reinforcement. For mycelium materials, the matrix is mycelium, and the reinforcement is the natural fiber. Mycelium surrounds the fibers and maintains their relative position inside the material. The fibers, in turn, largely influence the mechanical and physical properties of the composite [1]. A key aspect of improving the mechanical and physical properties of mycelium materials is the implementation of organic or inorganic particles [9,10,11]. Due to the wide variety of reinforcement particles, mycelium nanocomposites can potentially be designed for specific functions and applications, such as fire resistance and mechanical improvement.

Recent studies of such hybridization using glass improved the fire performance of mycelium materials as a result of significantly higher silica (inflammable) concentrations and low combustible material content [11]. It takes almost six times as much time for mycelium materials incorporating 50 wt.% glass fines to flash over as it takes synthetic materials, such as extruded polystyrene insulation foam, and two times as much as particleboard [11]. This study suggests that mycelium materials are very economical and exhibit far better fire safety parameters than the traditional construction materials tested (extruded polystyrene foam and particleboard made from wood flakes and bonded with moisture-resistant synthetic resin) [11]. However, a major problem with incorporating high contents of inorganic matter in the substrate might be the biocompatibility with white-rot fungi. Minerals limit the growth of the hyphae over poor nutritional surfaces and can therefore influence the bond between mycelium and the lignocellulosic fibers that must hold the material together.

To maintain sufficient mycelial growth, glass fines that comprise primarily silica (SiO_2_) and up to 30 wt.% organic surface matter were used in the particular research by Jones et al. [11]. Very limited research has further investigated the integration of additives in mycelium materials, with the exception of a study that indicates that the compressive strength of mycelium materials containing sand or gravel aggregates and wood chips increases up to 300% [12]. A patent makes note of the addition of components such as silica, clay and perlite to the fibers to retain moisture or enhance the viscosity of the substrate [13]. Thus far, no other study has investigated the fabrication, growth methods and mechanical properties of mycelium materials that incorporate inorganic (nano)particles.

Yet, nanotechnology and nanomaterials have great potential to improve the properties of different materials. Nanoparticles, like nanoclay, are widely used in various industries and areas of research, such as computing, adhesives, textiles, pharmaceutical and automotive [14,15,16]. For example, the reinforcement of particleboard and plywood panels with nanoSiO_2_, nanoAl_2_O_3_, and nanoZnO was reported to significantly decrease formaldehyde emission [17,18]. During the past decades, rapid developments have occurred in the area of polymer/clay nanocomposites. Most early studies focused on synthetic polymer, such as polyamides [18], polyimides [19], methacrylates [20,21] or polystyrene [22]. Nanoclay also offers an improved dimensional stability in wood–plastic composites [23]. Moreover, for wood–plastic composites, it is reported that flexural strength, tensile strength and elongation and water absorption are improved by the addition of nanoclay; the most interesting properties are observed in specimens with 5% of nanoclay content [24]. This improvement is due to the formation of bonds between the hydroxyl groups of nanoclay and the wood flour components. The addition of clay nanoparticles to cotton-based polymer composites resulted in increased char yield, therefore rendering them flame retardant [25].

One of the most common nanoclay forms is montmorillonite with a particle thickness of 1 nm, crosswise 70 to 100 nm [26,27]. Montmorillonite clays have a layered structure, and each layer is constructed from tetrahedrally coordinated Si atoms fused onto an edge-shared octahedral plane of either Al(OH)_3_ or Mg(OH)_2_ [26]. The layers exhibit excellent mechanical properties parallel to the layer direction due to the nature of the bonding between these atoms [26]. The principle for using nanoclay is to separate not only clay aggregates, but also individual silicate layers in a polymer [26]. The choice for montmorillonite nanoparticles is mainly motivated by their wide availability and inexpensiveness [15,28]. The main advantage is that a minimal content (1–5 wt.%) of such additives can improve the reinforcement of the polymer matrix [15,29,30]. Moreover, several studies have shown that the resulting organic–inorganic hybrids possess tremendous improvement in tensile strength and modulus, gas permeability, heat distortion temperature, and flammability [31].

This paper focuses on the incorporation of nanoparticles and the development of intricate gradients in the material’s arrangement. The goal of this work is to investigate the production of organic–inorganic hybrids and their material properties. The influence of nanoclay on the physical and mechanical properties of mycelium materials is studied. The hypothesis is that the hybridization of mycelium composites with nanoparticles improves mechanical properties as the nanoclay can reinforces the internal vessels of the lignocellulosic fibers. This is the first study undertaking a longitudinal analysis of nanoclay–mycelium hybrid materials by using different characterization methods, such as SEM, FTIR and mechanical testing.

**Figure 1:**
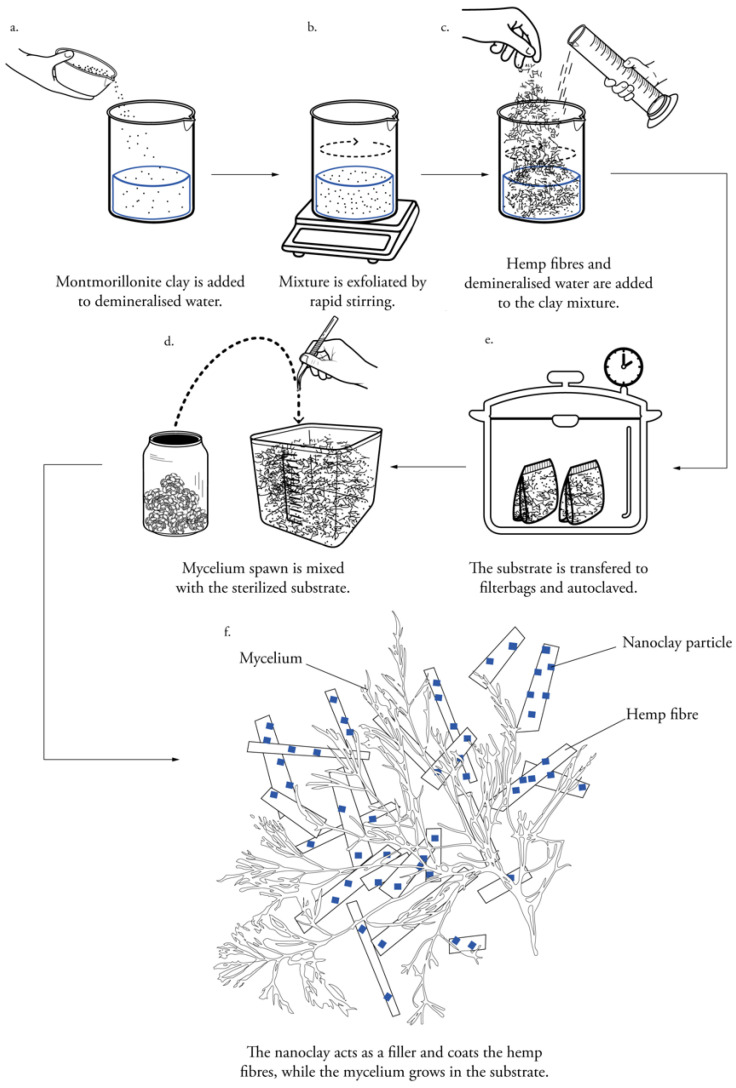
Schematic description of the fabrication process of nanoclay-reinforced mycelium composites.

The methodological approach focusses on the fabrication of nanoclay-coated fibers, inoculated with mycelium as binder (Figure 1). The experimental design was developed gradually and then combined in this work. The effect of nanoclay particles on the fabrication process and properties was mapped by analyzing the surface colonization rate, microscopic structure, chemical changes and mechanical properties.

## 2. Materials and Methods

### 2.1. Fungal Species

*Trametes versicolor* (M9912) spawn was purchased from Mycelia bvba (Veldeken 38A, 9850 Nevele, Belgium). The species were conserved on a grain mixture at 4 °C in a breathing Microsac 5 L bag (Sac O2 nv, Nevele, Belgium).

### 2.2. Materials

Studies were performed on the following fiber types: 5–25 mm hemp fibers (Aniserco S.A, Groot-Bijgaarden, Belgium). The superfine powder Ventoux montmorillonite clay was obtained from EMSPAC (Mons, France) in an untreated state.

### 2.3. Preparation of Nanoclay-Coated Substrate

Hemp nanocomposites containing 2.5 wt.% (weight percentage) montmorillonite clay as a filler material were prepared in batches of 820 g. The montmorillonite clay was exfoliated by rapid stirring for 30 min (ambient conditions) in 400 mL demineralized water, after which 25 wt.% hemp fibers and 72.5 wt.% ddH_2_O were mixed in a bigger flask (Figure 1). The fiber/clay substrate was autoclaved at 121 °C for 20 min. The bags were left to cool down for 24 h. Other studies filtered and washed the material in acetonitrile and deionized water [25,32]. We chose not to follow this procedure in this study due to the toxicity of acetonitrile.

### 2.4. Particleboard Fabrication for Bending and Tensile Testing

In a laminar flow hood, 10 wt.% of mycelium spawn was mixed with the sterilized fibers (Figure 1). During the first growth phase, the substrate grew in bags with a depth-filtration system that allowed for airflow. The bags were stored in an incubation room at 26 °C and relative humidity of 60%. The mycelium homogeneously colonized the substrate in chunks. The bags were kneaded every day to stimulate the strengthening of the mycelium. After 5 days, the substrate was crumbled by hand to re-activate the mycelium. It was then distributed in Microbox containers (purchased at SacO2, Deinze, Belgium) with a depth-filtration system on top (185 × 185 × 78 mm). After 12 days, the substrate had taken the shape of the rectangular molds. Subsequently, the samples were removed from the molds and incubated again for 5 days to achieve a homogeneous colonization on the sides that were previously in contact with the mold.

The samples were compressed with an Instron 5900R test bench that had an oven built around it. A maximum force of 30 kN was applied at 2 kN/min. When a displacement of 50 mm was reached, the load was kept constant for 1 h at a temperature of 200 °C.

The samples were heat-pressed to an aimed thickness of ±15 mm. Heat-pressed samples were dried in a convection oven at a temperature of 70 °C for 10 h, until the weight stabilized, and all humidity evaporated. The particle boards were then stored at 21 °C and 65% RH for 3–4 weeks before testing.

**Figure 2:**
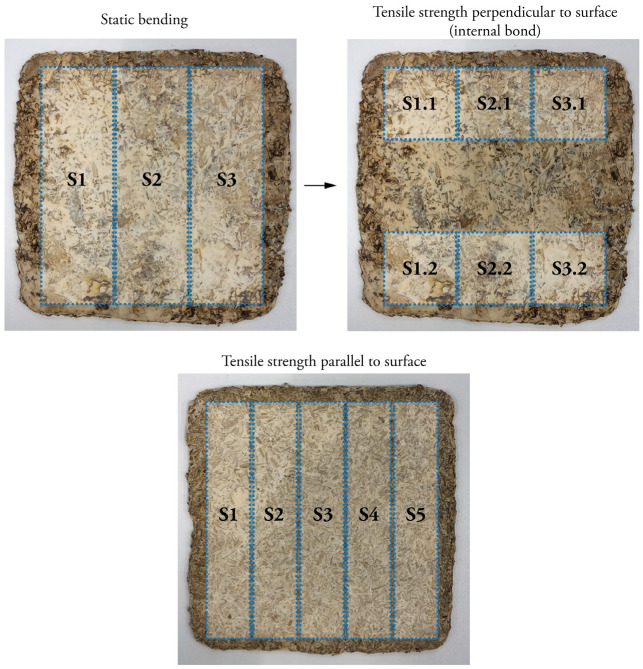
Example of the cutting pattern of specimens for the different mechanical tests.

Finally, the samples were cut with a thin blade saw, following the specimen dimensions set out by the several standards for mechanical tests (Figure 2). For the static bending tests, three specimens of 190 × 50 mm were cut from one particleboard. After loading, the non-damaged parts were reused for the tests on tensile strength perpendicular to the surface (internal bond) and cut to 50 × 50 mm. For tensile strength parallel to the surface, 5 specimens of 180 × 30 mm were cut from one particleboard.

### 2.5. Specimens for Compressive Testing

Compression tests were carried out with cylindrical specimens (h: 38 mm, d: 100 mm), whereby the quantity of nanoclay varied between 1 wt.%, 2.5 wt.%, 3 wt.% and 5 wt.%. The montmorillonite clay was exfoliated by rapid stirring for 30 min (ambient conditions) in demineralized water, after which hemp fibers and ddH_2_O were mixed in a bigger flask. The fiber/clay substrate was autoclaved at 121 °C for 20 min. The bags were left to cool down for 24 h. Similarly, as described above, 10 wt.% of mycelium spawn was mixed with the sterilized substrate and incubated in bags for 5 days, after which it was packed in cylindrical Microbox containers (purchased at SacO2, Deinze, Belgium). After 12 days, the samples were removed from the molds and incubated again for 5 days to achieve a homogeneous colonization on the sides.

### 2.6. Substrate Inoculation for Growth Studies

The substrate for the growth studies was also prepared in the same way as described above. The samples were composed of varying nanoclay quantities (between 1 wt.%, 2.5 wt.%, 3 wt.% and 5 wt.%). Inoculation was performed by applying a 4–5 mm fragment of grain spawn in the middle of the petri dish containing 15 g of humid hemp fibers. Each experiment was conducted with triplicate dishes. The petri dishes were inverted and incubated at 26 °C in darkness for 9 days.

### 2.7. Surface Colonisation Rate Measurements and Analysis

Daily growth was monitored every 6 to 12 h, after an initial growth of 24–72 h, using a CanoScan 9000F Mark II (Canon), with a pixel density of 300 dpi.

Analysis of the mycelium extension rate was done by measuring the surface area covered by the mycelium. The scanned images were imported in Fiji [33], the contrast and brightness were adjusted and the outline of the mycelium was traced by using the freeform or circle tool. The pixel-to-surface measurement tool in ImageJ was employed to convert this pixel tracing in quantitative results.

### 2.8. Scanning Electron Microscopy

To evaluate the microstructural geometry of the mycelium, Scanning Electron Microscopy (SEM) characterization was carried out using a JEOL JSM-IT300 microscope in low vacuum, which allowed pressure in the chamber of up to 20 Pa, using a 5 kV accelerating voltage. The specimens were mounted on aluminum stubs with double-sided tape. Images were taken with the Backscattered Electron Detector (BED) or Secondary Electron Detector (SED) at zoom of 500×, 1000× and 2000×. Samples were taken from the growth studies (see Section 2.6) grown for 22 days and air-dried for 5 days, at different locations in the Petri dish (5 replicates).

### 2.9. Fourier Transform Infrared Spectroscopy

Chemical characterization was performed for 1–2.5–3.5–5% nanoclay mycelium. For the Fourier Transform Infrared (FTIR) spectroscopy, all IR spectra were acquired on a Nicolet 6700 FT-IR spectrometer from Thermo Fischer Scientific (Waltham, MA, USA). The FTIR instrument is equipped with an IR source, DGTS KBr detector and KBr beam splitters and windows. FTIR spectra are recorded in single bounce Attenuated Total Refractance (ATR) mode using the Smart iTR accessory, equipped with a diamond plate (42° angle of incidence). The spectra were recorded with automatic atmospheric correction for the background. All samples were measured at a spectral resolution of 4 cm^−1^, with 64 scans per sample. For every species, three biological replicate specimens were analyzed, and five recordings of each sample were performed to ensure the reproducibility of obtained spectra. The processing of the spectra, such as calculating the average, the peak height and area, was done using Spectragryph v1.2.8 software. The spectra were cut between 4000 and 600 cm^−1^ bands, followed by the construction of a linear baseline. Finally, the peaks were normalized for peak maxima.

Samples were taken from the growth studies (see Section 2.6) grown for 22 days and air-dried for 5 days, at different locations in the Petri dish (5 replicates). These samples were placed in microcentrifuge tubes and then in a TissueLyser II from QIAGEN (Hilden, Germany) with a small metallic ball. The resulting fine powder was mounted on the FTIR.

### 2.10. Determination of Bending Behaviour

Since no standard exists for testing mycelium materials, three-point static flexural tests were performed according to specifications of norms that were expected to result in similar properties. The characteristics of mycelium materials are situated somewhere between foam and wood-based panels; therefore, we refer to the following standards: ISO 16978—*Wood-based panels—Determination of modulus of elasticity in bending and of bending strength* [34] and ISO 12344—*Thermal insulating products for building applications—Determination of bending behavior* [35]. According to ISO 16978, “the test pieces shall be rectangular, the width shall be 50 mm, the length shall be 20 times the nominal thickness plus 50 mm (minimum 150 mm).” On the other hand, ISO 12344 states that “specimens shall have a width of 150 mm and a length of 5 times the nominal thickness plus 50”. For the mycelium samples, according to the first standard, the specimen dimensions should have been 350 × 50 × 15 mm or 150 × 50 × 4 mm. Following the second standard, the specimen dimensions should have been 125 × 150 × 15 mm or 70 × 150 × 4 mm. The test specimen dimensions were 170 × 50 mm. The distance between the supports was 150 mm for all tests. A loading speed of 2.5 mm/min was applied. The samples were tested using an Instron 5900R load bench with a load cell of 10 kN. The bending strength ${ƒ}_{m}$ of each test piece is calculated from the formula [34]:

(1) $${ƒ}_{m}=\frac{3F_{\max}l_{1}}{2{\mathrm{bt}}^{2}}\;\left[\mathrm{MPa}\right]$$

where F_max_ is the maximum load [N/mm^2^], l_1_ is the distance between the centers of the supports [mm], b is the width of the test piece [mm] and t is the thickness of the test piece [mm].

The modulus of elasticity E_m_, is calculated from the formula [34]:

(2) $$E_{m}=\frac{l_{1}^{3}\left(F_{2}-F_{1}\right)}{4{\;\mathrm{bt}}^{3}\left(a_{2}-a_{1}\right)}\;\left[\mathrm{MPa}\right]$$

where l_1_, b and t are the dimensions as defined above, F_2_ − F_1_ is the linear portion of the load-deflection curve [N], F_1_ is 10% and F_2_ is 40% of the maximum load. The term a_2_ − a_1_ represents the increment of deflection at the mid-length of the test piece (corresponding to F_2_ − F_1_).

### 2.11. Determination of Tensile Behaviour Parallel to the Surface

Tensile strength parallel to the surface was measured according to ASTM 1037—*Standard Test Methods for Evaluating Properties of Wood-Base Fiber and Particle Panel Materials* [36]. The specimen dimensions were 170 × 30 mm. A loading speed of 1 mm/min was applied. Five samples of each treatment were tested using an Instron 5900R load bench with a load cell with a maximal capacity of 10 kN.

The specific strength and modulus were calculated using the following formulas:

(3) $$T_{\sigma }=\frac{\sigma_{u}}{\rho }\;\left[\mathrm{kN}\cdot m/\mathrm{kg}\right]$$

and

(4) $$T_{E}=\frac{E}{\rho }\;\left[10^{6}\;m^{2}\;s^{-2}\right]$$

where $T_{\sigma }$ is specific tensile strength (kN·m/kg or or MPa/(g/cm^3^)), $\sigma_{u}$ is ultimate tensile strength (MPa), ρ is density (g/cm^3^), T_E_ is specific Young’s modulus (10^6^ m^2^ s^−2^ or GPa/(g/cm^3^)) and E is Young’s modulus (GPa).

### 2.12. Determination of Tensile Behaviour Perpendicular to the Surface

This test was performed to determine the cohesion (internal bond) of the material. The test was performed according to *EN 319:1993 Particleboards And Fiberboards. Determination Of Tensile Strength Perpendicular To The Plane Of The Board* [37]. The specimens with dimension 50 × 50 mm were glued on aluminum loading blocks. After 24 h curing, the block was mounted into the grips. A loading speed of 0.5 mm/min was applied. The specimens were loaded at a uniform motion rate until failure occurred. Two samples of each treatment were tested using Instron 5900R load bench with a maximal capacity of 10 kN.

### 2.13. Determination of Compressive Behaviour

Compressive stiffness was determined following ISO 29469—*Thermal insulating products for building applications—Determination of compression behavior* [38] on an Instron 5900R load bench with a 100 kN capacity and a 10 kN load cell at ambient conditions (25 °C and ~50% RH). The 10 kN load cell was used in order to obtain the most accurate results, since low ultimate loads values were expected. The tests were displacement controlled with a rate of 5 mm/min. The contact surface was not perfect due to the rough surfaces of the samples. The test was stopped when a fixed strain was reached in the specimen, varying between 70% and 80%. The mechanical compressive stiffness or Young’s modulus was obtained from the first slope of the stress–strain curve with the tangent modulus.

### 2.14. Statistical Analysis

The data were statistically analyzed in Microsoft Excel and graphed with GraphPad Prism (version 8.1.2). Data were checked for normality (*p* ≥ 0.05) using a Kolmogorov–Smirnov test. An one-way analysis of variance (ANOVA) was used for normal data, and significant differences were considered at *p* ≤ 0.05. The multiple comparisons test for normal data was generated based on Tukey’s family error rate. For non-parametric data, the Kruskal–Wallis test was conducted, and significant differences were considered at *p* ≤ 0.05. The Dunn’s multiple comparison test was used for the non-parametric data.

## 3. Results

As this work deals with the fabrication of nanoclay-reinforced mycelium composites, it focused on the dispersion of inorganic nanoparticles in a hemp substrate as well as on mechanical performance. Particleboards were manufactured and mechanically tested to obtain first flexural modulus and strength, then elastic modulus and strength parallel and perpendicular to the surface.

### 3.1. Mycelium Growth and Surface Colonisation

**Figure 3:**
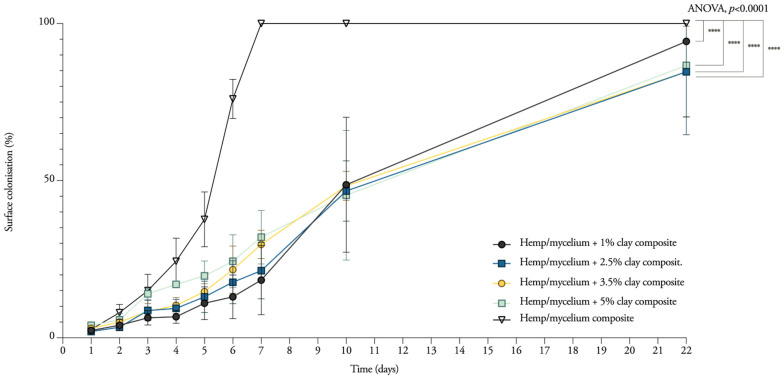
Radial mycelium extension rate measured as surface colonized (%) over 22 days for *T. versicolor* (M9912) on a hemp and 1–5% nanoclay substrate.

The fungus used in this research is a white-rote species. Lignocellulose is the primary source of nutrient. Hence, the added minerals might limit the growth of the hyphae if the fibers are coated with clay particles. To assess the influence of the amount of nanoclay on the growth kinetics of mycelium, different proportions were mixed with hemp fibers. Remarkably, the results suggest that composites containing 5 wt.% of nanoclay grow 5% faster than composites with 1 wt.% of nanoclay during the first 7 days (Figure 3). The *T. versicolor* mycelium stays concentrated in the middle of the Petri dish during the first 7 days, and on the 10th day, only small, thin white branches start to explore the rest of the substrate. The graph gives an image of the growth rate, which shifted from 5 wt.% nanoclay having the highest rate after 7 days to 1 wt.% nanoclay having the highest rate after 22 days. After 10 and 22 days, composites with 1 wt.% fully colonized the Petri dish before the other compositions. The initial exploring hyphae slowly covered the whole substrate.

**Figure 4:**
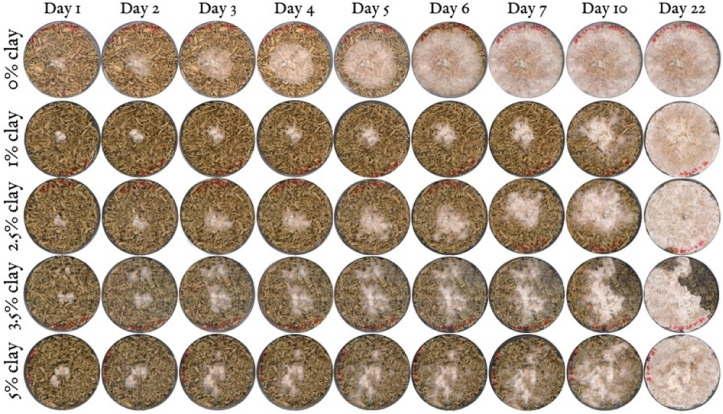
Colonization over 22 days for *T. versicolor* (M9912) on a hemp and 0–5% nanoclay substrate.

As expected, the nanoclay composites grew significantly slower than samples without nanoclay (two-way ANOVA, adjusted *p* < 0.0001). Samples containing nanoclay grew 24% to 29% slower than the control samples. The slower growth of mycelium makes the substrate more prone to contaminations. From the three replicates, at least one sample containing 3.5 wt.% and 5 wt.% was partially contaminated, blocking the hyphae from fully colonizing the rest of the substrate (Figure 4). Moreover, the hydrophobic nature of clay forms a barrier to the uptake of moisture and oxygen. In accordance with the present results, previous studies have demonstrated that the presence of nanoclay makes the wood–plastic composites less accessible to the fungus due to the reduction in oxygen content and moisture uptake, and nutrient shortage [39,40].

### 3.2. Morphological Analysis of Hyphae and Nanoclay

**Figure 5:**
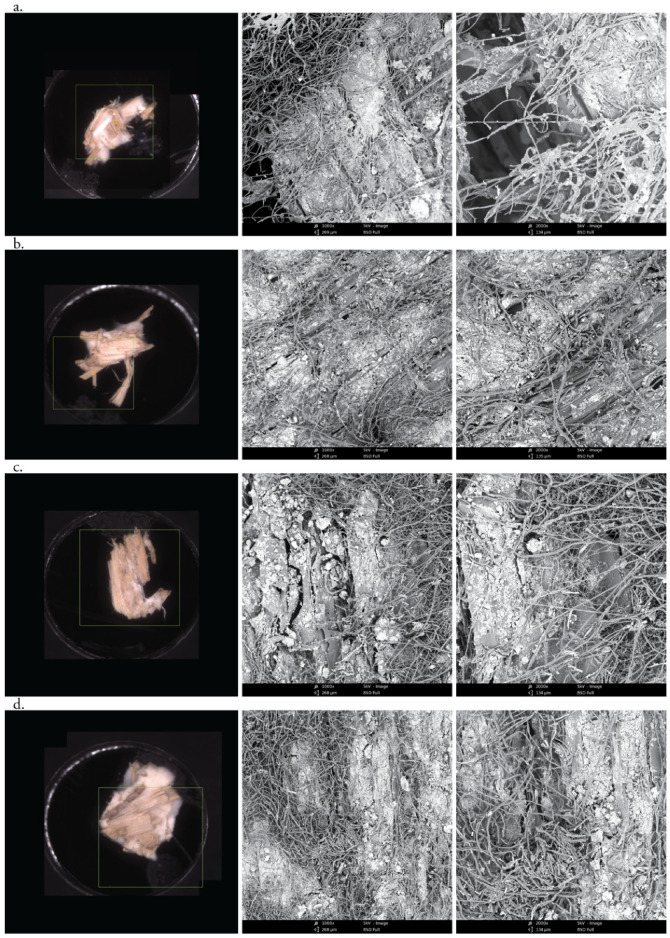
Morphological characterization by SEM images of *T. versicolor* (long threads) on a hemp (dark-gray, plate-like fibers) and nanoclay (light-gray particles) substrate after 7 days. (**a**) 1 wt.% nanoclay. (**b**) 2.5 wt.% nanoclay. (**c**) 3.5 wt.% nanoclay. (**d**) 5 wt.% nanoclay. Micrographs were taken at zoom 10×, zoom 1000× and zoom 2000×.

**Figure 6:**
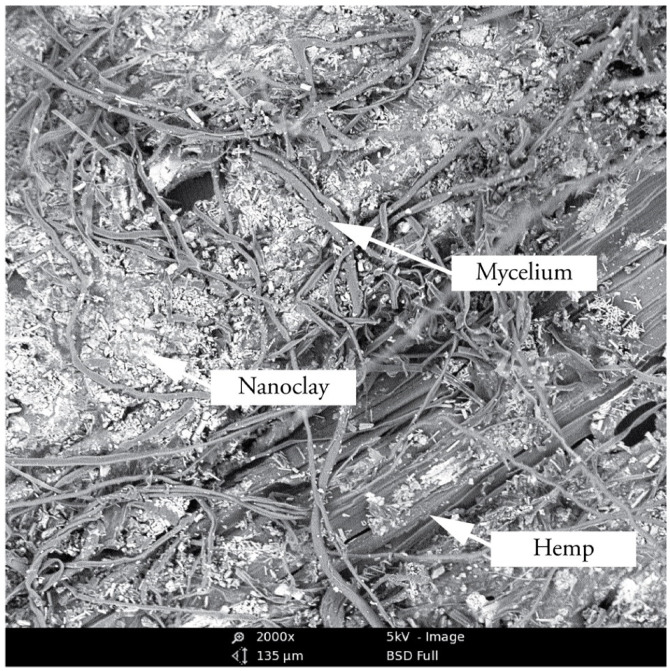
Micrograph of 2.5 wt.% nanoclay-mycelium composite showing the hyphae that grow inside the nanoclay, which is coated around the hemp fibers.

To further investigate the interactions between the organic and inorganic substrate with mycelium, different mixtures (1 wt.%, 2.5 wt.%, 3.5 wt.% and 5 wt.% nanoclay) were dried after having grown for 22 days and observed by SEM (Figure 5). The images show a distribution of nanoparticles around the hemp fibers. The amount of nanoclay (between 1 and 5%) does not seem to alter the ability of hyphae to grow in and around the fibers. The hyphae were able to penetrate the clay particles, as their growth tracks are marked in the clay (Figure 6). Interestingly, some nanoclay particles were observed around the hyphae. It is possible that the distribution of particles was altered while handling the samples, but the nanoparticles could also have been transported and moved during the growth of the hyphae. Fibers with 1 and 2% nanoclay were only slightly coated by the particles, while samples with 3.5 and 5% nanoclay were packed with particles that reached into the fibers’ cell-wall structure. Still, the concertation of hyphae was higher in cavities and places where the fibers were not covered by nanoclay.

### 3.3. Spectral Response to Nanoclay Concentrations

**Figure 7:**
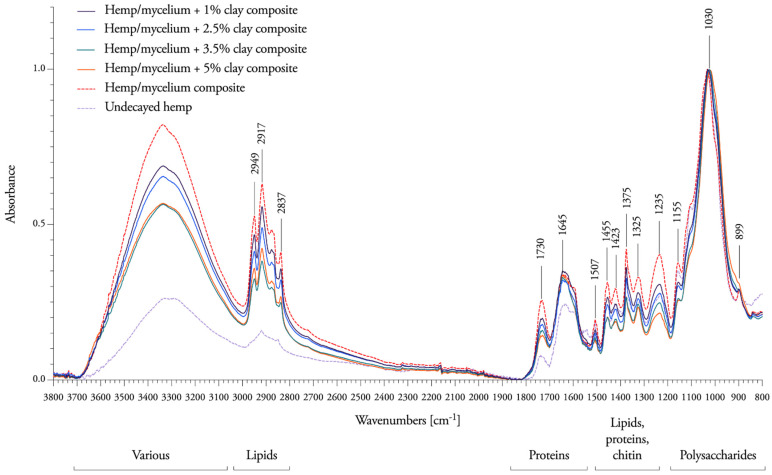
Mean FTIR spectra of 1–2.5–3.5–5% nanoclay–mycelium composites, mycelium composite without nanoclay (dotted line) on a hemp substrate after growth of 7 days, and undecayed hemp fibers (dashed line).

Next, to understand the influence of different concentrations of nanoclay on the degradation capacity of *T. versicolor*, FTIR spectra were compared with undecayed hemp fibers and mycelium-hemp composites (Figure 7). For an exhaustive chemical analysis of the degradation between decayed and undecayed fibers, we refer to Elsacker et al. 2019 [1]. Here, we focus only on the impact of the nanoclay filler on the degradation capacity in the “fingerprint” region between 1800 and 600 cm^−1^.

Mycelium-hemp composite samples showed the presence of bands at 3310 cm^−1^ for O-H stretching of bonded hydroxyl groups, 2949 and 2837 cm^−1^ for C-H stretching, 1731 cm^−1^ for C=O stretching, 1645 cm^−1^ for H-O-H deformation vibration of absorbed water and conjugated carbonyl groups, mainly originating from lignin, 1456 cm^−1^ for CH_2_ deformation vibrations in lignin and xylan, 1423 cm^−1^ for aromatic skeletal vibrations combined with C-H in plane deformation and for +C-H deformation in lignin and carbohydrates, 1156 cm^−1^ for C-O-C vibration in cellulose and hemicelluloses, 1032 cm^−1^ for C=O stretching vibration in cellulose, hemicelluloses and lignin and 1000−800 cm^−1^ for C−H bending vibration in cellulose.

**Figure 8:**
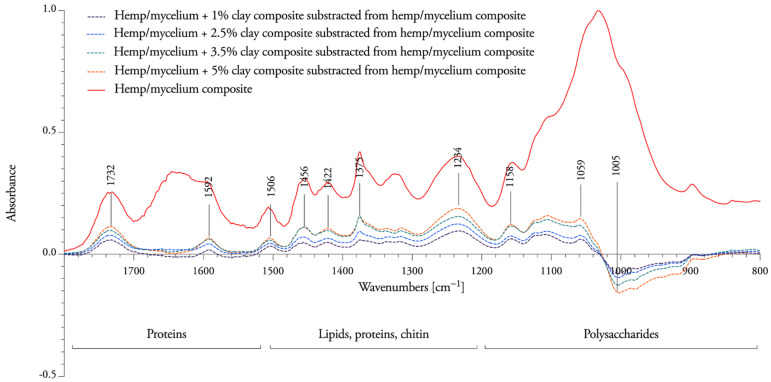
The subtracted peaks of nanoclay–mycelium composite (dotted line) from mycelium composite fibers without clay (solid line) below the baseline show an appearance of new bands due to degradation by the fungi, while the subtracted peaks above the baseline show the decrease of bands. The intensities are around zero when there is no change in the chemical composition of the fibers during the mycelium interaction.

The intensities of all peaks of nanoclay composites decreased slightly between 1800 cm^−1^ and 1021 cm^−1^, which indicates that *T. versicolor* has more difficulty accessing and decaying the hemicellulose and lignin when the amount of nanoclay increases. New bands were formed at 1005 cm^−1^, revealing that cellulose is mostly resistant to degradation when nanoclay is present in the composite (subtracted peaks under the baseline in Figure 8). No changes in chemical composition were observed at 1645 cm^−1^ (H-O-H deformation vibration of absorbed water and conjugated carbonyl groups, mainly originating from lignin), or at 899 cm^−1^ (C−H bending vibration in cellulose). The percentage of nanoclay is visible on the spectral response as fibers containing 1% nanoclay are more degraded than the ones containing 5%. This suggests that nanoclay obstructs the nutritional access to the fibers.

### 3.4. Flexural Properties of Nanoclay-Mycelium Particleboards

Foremost, the goal of this study was to investigate the influence of the addition of nanoclay particles on the mechanical properties of mycelium composites. The particleboards were all visually different, showing variations in the growth pattern of the organism. These fungal morphological changes are associated with extracellular signaling and metabolic pathways [41].

**Figure 9:**
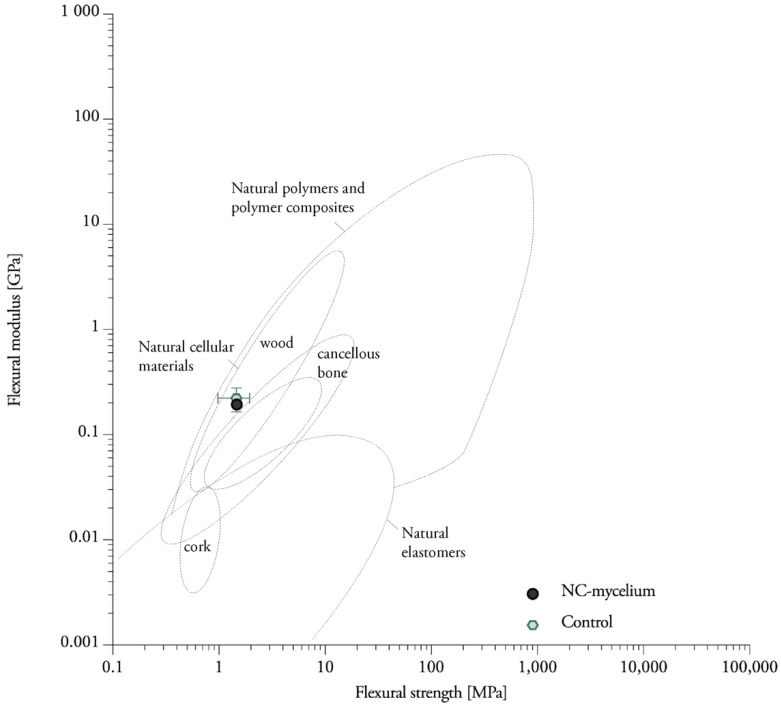
A material property, plotting Young’s modulus against strength for nanoclay–mycelium composites on an Ashby chart for natural materials.

The addition of nanoclay to the fibers did not significantly change the bending behavior of mycelium composites (Table 1). The control samples without nanoclay (1.46 MPa) are situated close to the NC-mycelium samples (1.47 MPa). The samples tested within this study displayed flexural properties that can be related to those of natural materials such as wood, cork and cancellous bone (Figure 9) [42]. It is not surprising that mycelium materials are efficient when bending, because natural materials such as wood, cork and bamboo also indicate high values for the flexure index [42]. Similarly, these natural cellular materials have low densities due to the high volume of voids.

### 3.5. Tensile Properties Parallel to the Surface

**Figure 10:**
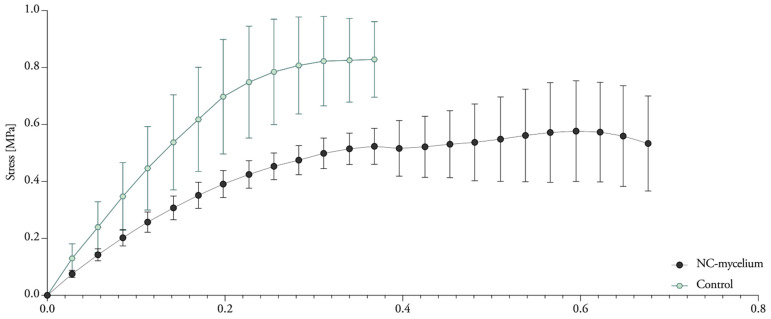
Stress–strain curves of tensile tests of nanoclay–mycelium composites.

The composites show ductile behavior under tension, with the failure occurring due to the breakage of the mycelium binding between fibers (Figure 10). The values of tensile strength and elastic modulus of NC-mycelium are lower (0.62 MPa) than those of the control samples without nanoclay particles (1.14 MPa) (Table 2). Although pneumatic grips with a cardboard lining were used to avoid failure before testing, the stress concentrations and failure developed for some replicates in the clamping region. Other studies show that analysis of specimens that failed at the grip–specimen interface versus those that failed at mid-substance showed no significant difference in their tensile properties [43]. However, these data must be interpreted with caution, and further studies are recommended.

### 3.6. Tensile Properties Perpendicular to the Surface

**Figure 11:**
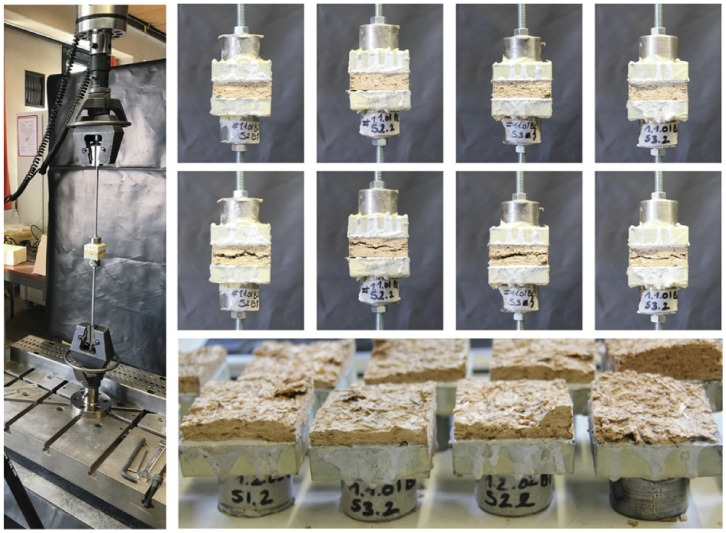
Detail of specimens and fixtures before and after loading for tension tests perpendicular to the surface (internal bond).

**Figure 12:**
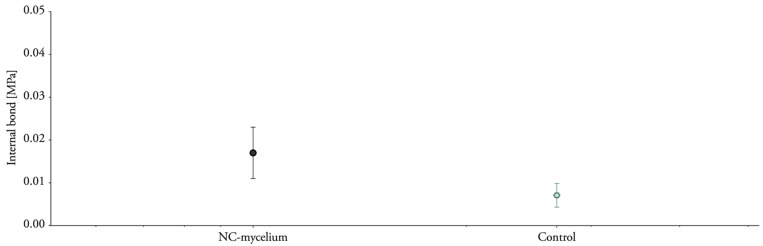
Internal bond strength of nanoclay–mycelium composite. Labels with different letters indicate a statistical difference (*p* < 0.05) among the specimens.

Samples were submitted to tension perpendicular to the surface in order to quantify the adhesion of the fibers (Figure 11). The results (Table 3) indicate higher internal bonding for nanoclay-based mycelium composites (0.023 MPa) compared to the control sample (0.007 MPa). These results contrast with the previous tests and might be related to the orientation of the specimen during testing. The samples were cut from a particleboard and teared apart in the opposite direction from the tensile tests described in the previous section. If the materials were grown directly in the geometry of the specimen, the mycelium layer, which grows at the surface of the materials, would probably impact the internal bond. Additionally, the composites do not meet the requirements for lightweight particleboards (0.1 MPa) as established by EN 622-3 (Figure 12).

### 3.7. Compressive Properties

**Figure 13:**
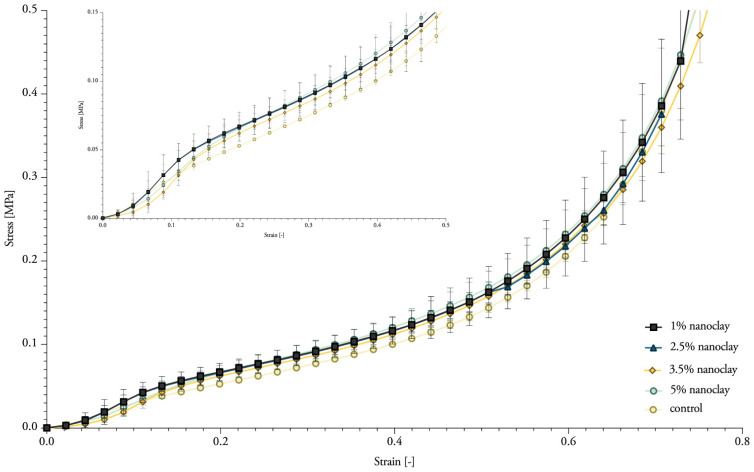
Typical stress–strain curve of compression for mycelium composites grown with 1 to 5% nanoclay, showing a zoom on the first straight portion of the curve. The standard deviation is performed with 3 specimens (mean ± one standard deviation).

**Figure 14:**
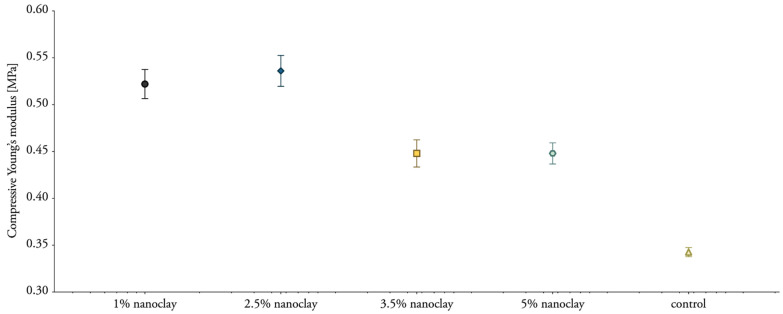
Compressive Young’s modulus of mycelium composites during uniaxial compression with 1 to 5% nanoclay. The standard deviation is performed with 3 specimens (mean ± one standard deviation).

Although nanoclay was expected to influence the compressive strength, it did not significantly (Table 4). The samples showed progressive load-deflection behavior (Figure 13). Mechanical compressive stiffness was obtained from the slope (first distinct straight portion) of the stress–strain curve with the tangent modulus. The compressive stiffness ranged between 0.45 and 0.54 MPa for nanoclay-reinforced composites, compared with 0.34 Mpa for mycelium composites without nanoclay (Figure 14). The values of the compressive Young’s modulus range in the same order as previously reported compressive properties for mycelium composites [1]. Yet, we expected to see a more pronounced fluctuation in the results with the addition of different percentages of inorganic particles.

## 4. Discussion

This section discusses the mechanical properties of mycelium materials and nanoclay–mycelium materials and gives an overview of values obtained in other investigations.

### 4.1. Nanoclay-Mycelium Materials Meet the Requirements of Softboards for the Flexure Index

**Figure 15:**
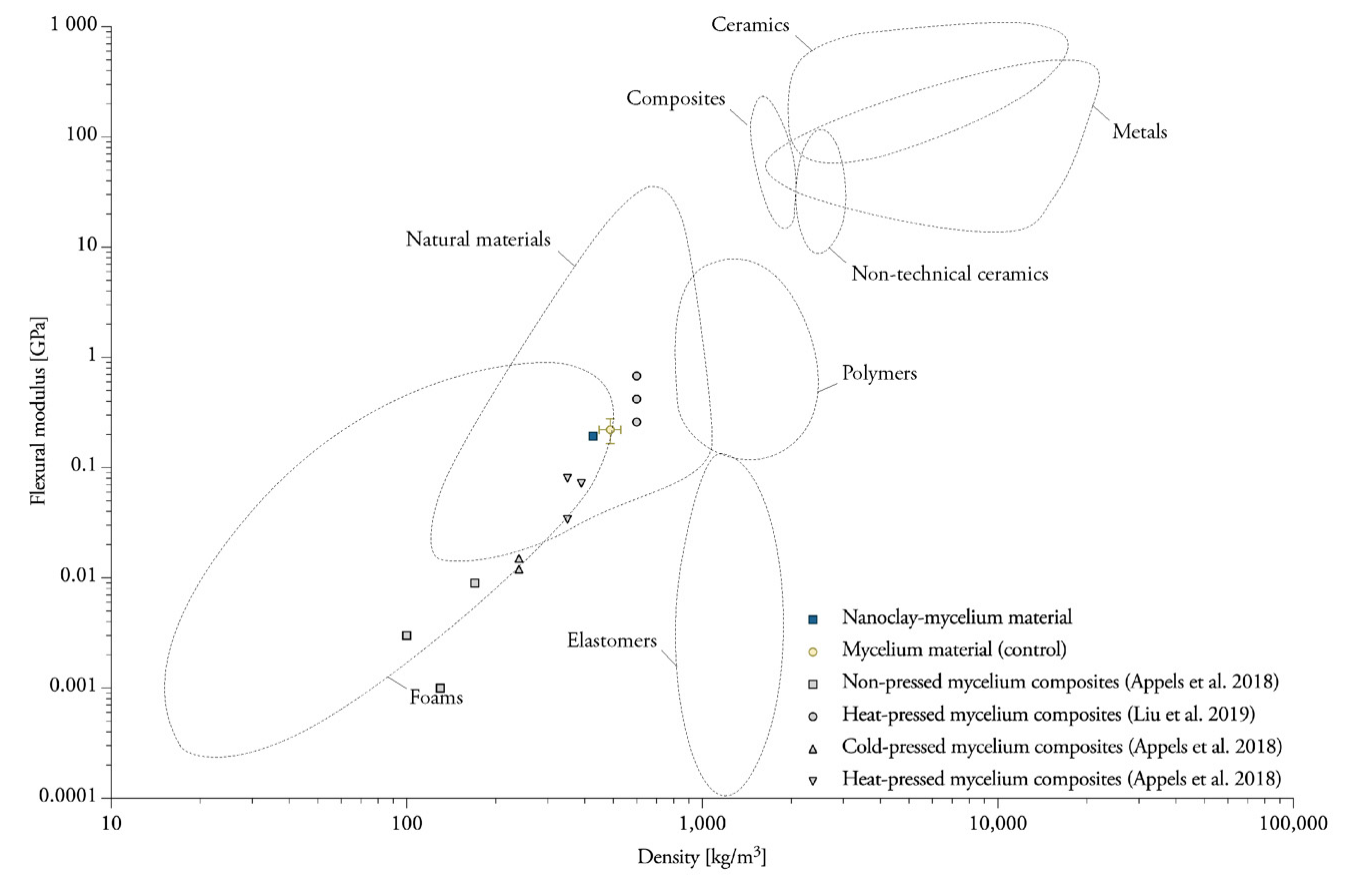
Overview of data on the flexural modulus for mycelium materials obtained in this and other studies. Data compiled from [3,44]. Plotted on Ashby chart for engineering materials [57].

Nanoclay–mycelium materials meet the requirements of bending for softboards as defined in EN 622-4 (2019) (type SB.LS) in dry, humid, and exterior conditions and load-bearing use. The findings combined extend some of the existing data on the flexural properties for mycelium materials obtained in other studies (Figure 15). The flexural strength of heat-pressed rapeseed and cotton mycelium materials ranges between 0.62 and 0.87 MPa. The flexural modulus of the same samples ranges between 0.03 and 0.07 GPa [3]. However, researchers were able to achieve higher flexural modulus (1.4–4.6 MPa) with mycelium–cotton stalk composites under 160, 180 and 200 °C pressing temperatures [44]. Heat-pressing was done with a pressure of 3.5–4.0 MPa to a density of 600 kg/m^3^ [44], while the density of the previous research was only 350 kg/m^3^ [3] and average density of samples in this research was 363.18 kg/m^3^. Non-pressed cotton plant materials were reported to have flexural strengths in the range of 0.007–0.026 MPa (These data must be compared with caution, because it was normalized by the authors to a standard polystyrene density) [45]. One other study on non-pressed mycelium materials made from crop residues and coated with edible films (carrageenan, chitosan and xanthan gum) reports a flexural strength of 0.01 MPa [46]. The flexural modulus ranges between 66.14 and 71.77 MPa for cotton and hemp mycelium materials [47]. A higher porosity in the discussed studies’ material strongly contributes to lower flexural strength, while a high density achieved by heat-pressing can further enhance the material properties.

The amount of existing literature on the mechanical properties of polymer-based montmorillonite nanoclay composites points to the great attention paid to this in academic and industrial sectors. For example, the flexural strength of wood flour–PLA composites with 0.5% sodium-montmorillonite clay was 13% larger than the value for the control sample without nanoclay [48]. In this thesis, flexural strength increased by only 1% for nanoclay-infused materials.

### 4.2. Heat-Pressing Is a Major Factor in Increasing Tensile Properties

**Figure 16:**
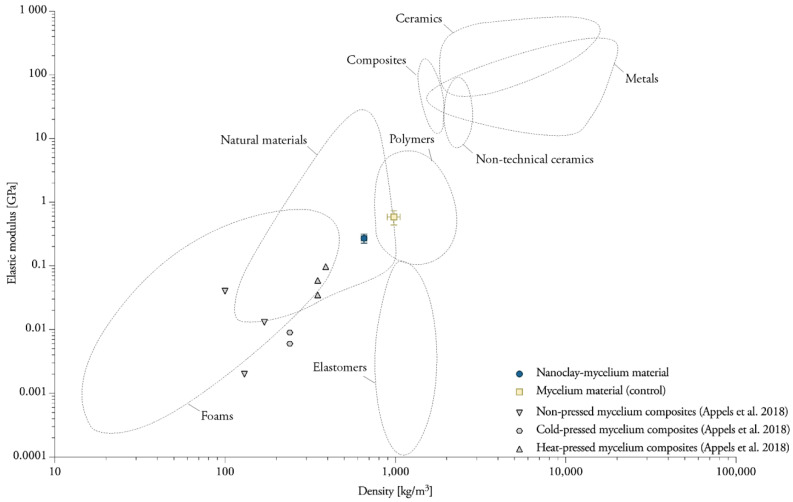
Overview of data about the elastic modulus for mycelium materials obtained in this and other studies. Data compiled from [3]. Plotted on Ashby chart for engineering materials [57].

Tensile strength (σ) of pure *Ganoderma lucidum* and *Pleurotus ostreatus* mycelium biofilms ranges between 0.7 and 1.1 MPa [5]. For pure wild types of *S. commune* biofilms, tensile strength ranges between 5.1 and 9.5 MPa, while for modified strains (deletion of the hydrophobin gene sc3), tensile strength increases to 15.6–40.4 MPa [49]. Reported tensile properties of composite mycelium materials vary for different types of feedstocks (Figure 16), and are for example higher for sawdust substrates (σ 0.05 MPa) than for straw substrates (σ 0.01–0.04 MPa) [3]. Heat-pressing was reported to be the major factor in increasing tensile strength and modulus (σ 0.13–0.24 MPa, E 35–97 MPa) in comparison to cold-pressing (σ 0.03 MPa, E 6–9 MPa) or non-pressing (σ 0.01–0.05 MPa, E 2–13 MPa) [3]. In comparison, the developed samples and conducted experiments in this research resulted on average in tensile strength that was six times higher (σ 1.14 MPa, E 585.98 MPa). A possible explanation for the divergence of these findings with the literature may the use of different production and testing methods, as there is no adequate standard for the production and testing of mycelium materials.

Tensile strength of wood flour–PLA composites slightly improves by 4% with the addition of montmorillonite clay [48]. According to the authors, the higher strength of the composites is caused by the intercalated structure of montmorillonite in the wood flour cell wall [48]. In addition, incorporation of nanoclay reduces the polarity of natural fiber and benefited the interface between the fiber and the PLA matrix [50]. The intercalated montmorillonite in polymer matrix improves the capability to transfer load from the polymer to the strong montmorillonite [51].

### 4.3. Mycelium Composites Have a Low Internal Bond

Other studies found an internal bond strength between 0.05 and 0.18 MPa, with mycelium–cotton stalk composites under 160, 180 and 200 °C pressing temperatures [44]. In a follow-up study, the same authors improved the internal bond to 0.34 MPa by immersing the materials in water until an uptake of 30% before heat-pressing. In comparison with that study, the internal bonding results presented in this work are very low: 0.023 MPa for nanoclay-based mycelium composites and 0.007 MPa for mycelium composites without nanoclay. Nonetheless, no other satisfying results were achieved by other researchers, as for example Sun et al. note that the composites were too weak to be measured in the internal bond strength test [52]. The values for the internal bond strength of the wood- and resin-based boards can significantly increase with the addition of 1–5% nanoclay [53,54]. The authors observed that the increase in brittleness due to the addition of nanoclay inclusions was balanced by the increase in ductility due to the higher press temperature. Previous studies reported that the maximum internal bond value (1.99 MPa) was obtained for particleboard with 2% nanoclay loading, which accounted for an improvement of 18.03% compared to the reference board [55]. The improved behavior can be explained by the percolation theory, which is applied to the networking capability of the clay nano-platelets [56].

## 5. Conclusions

The effects of hybridization of mycelium composites with nanoclay were evaluated in terms of morphological, chemical, and mechanical properties. Nanoclay causes a decreased growth rate, which appears to be up to 29% slower than that of control samples. Moreover, the slower growth of mycelium makes the substrate prone to contaminations. The distribution of clay particles was visualized through SEM imagery. The particles can penetrate into the fibers’ cell-wall structure, and some nanoclay particles were observed around the hyphae. The FTIR study demonstrated that *T. versicolor* has more difficulty accessing and decaying the hemicellulose and lignin when the amount of nanoclay increases. It seems that cellulose is more difficult to degrade when nanoclay is present in the composite. The ability of the fungus to degrade the nanoclay-coated fibers decreases with the increase of nanoclay.

The addition of nanoclay to the fibers does not significantly affect the bending behavior of mycelium composites. Tensile strength and elastic modulus of nanoclay–mycelium materials are lower than those of the control samples without nanoclay particles. While nanoclay enhances the properties of polymer composites, the hybridization with mycelium composites was not successful. On the other hand, strength values are higher than most of the existing data about flexural and tensile properties for mycelium materials obtained in other studies. The results indicate a slightly higher but negligible internal bonding and compressive stiffness for nanoclay-based mycelium composites.

The challenges of the hybridization of mycelium materials with inorganic reinforcements have become clearer. In general, this work provides a useful method for combining renewable organic sources, such as natural fibers and fungal mycelium, with nanoparticles. There is still a long way to go to understand all biological and engineering mechanisms needed to achieve specific structural properties using nano-mycelium materials.

## Figures and Tables

**Table 1:** Overview of the material properties in three-point bending of nanoclay–mycelium composites. The standard deviation is performed with triplicate specimens (mean ± one standard deviation).

Label	Dry Density [kg/m^3^]	Flexural Strength [MPa]	Flexural Modulus [GPa]
NC-mycelium hemp composite	426.98 ± 9.19	1.47 ± 0.02	0.19 ± 0.002
Mycelium hemp composite (control)	488.89 ± 41.09	1.46 ± 0.48	0.22 ± 0.06

**Table 2:** Overview of the material properties in tension of nanoclay–mycelium composites. The standard deviation is performed with triplicate specimens (mean ± one standard deviation).

Label	Dry Density [kg/m^3^]	Ultimate Tensile Strength [MPa]	Specific Tensile Strength [kN·m/kg]	Elastic Modulus [GPa]	Specific Modulus [10^6^ m^2^ s^−2^]
NC-mycelium hemp composite	654.55 ± 24.39	0.62 ± 0.10	0.95 ± 0.17	0.27 ± 0.04	0.42 ± 0.08
Mycelium hemp composite (control)	980.67 ± 84.77	1.14 ± 0.13	1.15 ± 0.07	0.59 ± 0.15	0.61 ± 0.17

**Table 3:** Overview of the material properties in internal bond of nanoclay–mycelium composites. The standard deviation is performed with 9 specimens (mean ± one standard deviation).

Label	Dry Density [kg/m^3^]	Ultimate Strength [MPa]	Specific Strength [kN·m/kg]	Elastic Modulus [GPa]	Specific Modulus [10^6^ m^2^ s^−2^]
NC-mycelium	340.85 ± 2.09	0.017 ± 0.006	0.049 ± 0.017	0.003 ± 0.000	0.009 ± 0.001
Control	492.32 ± 45.40	0.007 ± 0.003	0.01 ± 0.006	0.005 ± 0.002	0.01 ± 0.0007

**Table 4:** Overview of material properties in compression of mycelium composites grown with 1 to 5% nanoclay. The standard deviation is performed with three specimens.

Label	Dry Density [kg/m^3^]	Compressive Strength [Mpa]	Corresponding Strain [%]	Young’s Modulus [Mpa]
1% NC-mycelium	180.14 ± 0.01	0.12354	42.85	0.52 ± 0.02
2.5% NC-mycelium	183.16 ± 0.08	0.12356	42.52	0.54 ± 0.02
3.5% NC-mycelium	176.82 ± 0.02	0.11932	42.41	0.45 ± 0.01
5% NC-mycelium	175.31 ± 0.02	0.12804	43.00	0.45 ± 0.01
Control	172.34 ± 0.04	0.10715	43.50	0.34 ± 0.01

## Data Availability

Data is available upon request.
